# Mobile Phone Usage Detection by ANN Trained with a Metaheuristic Algorithm †

**DOI:** 10.3390/s19143110

**Published:** 2019-07-14

**Authors:** Efrain Mendez, Alexandro Ortiz, Pedro Ponce, Juan Acosta, Arturo Molina

**Affiliations:** School of Engineering and Sciences, Tecnologico de Monterrey, Mexico city 14380, Mexico

**Keywords:** artificial neural network, nanotechnology, optimization, sensors

## Abstract

Artificial neural networks (ANN) are widely used to classify high non-linear systems by using a set of input/output data. Moreover, they are trained using several optimization methodologies and this paper presents a novel algorithm for training ANN through an earthquake optimization method. Usually, gradient optimization method is implemented for the training process, with perhaps the large number of iterations leading to slow convergence, and not always achieving the optimal solution. Since metaheuristic optimization methods deal with searching for weight values in a broad optimization space, the training computational effort is reduced and ensures an optimal solution. This work shows an efficient training process that is a suitable solution for detection of mobile phone usage while driving. The main advantage of training ANN using the Earthquake Algorithm (EA) lies in its versatility to search in a fine or aggressive way, which extends its field of application. Additionally, a basic example of a linear classification is illustrated using the proposal-training method, so the number of applications could be expanded to nano-sensors, such as reversible logic circuit synthesis in which a genetic algorithm had been implemented. The fine search is important for the studied logic gate emulation due to the small searching areas for the linear separation, also demonstrating the convergence capabilities of the algorithm. Experimental results validate the proposed method for smart mobile phone applications that also can be applied for optimization applications.

## 1. Introduction

The economic and social benefits in nanotechnology have brought significant *research and development* (R&D) to governments and industries worldwide for two decades [1]. The principal areas of interest in nanotechnology are advanced materials, catalysts, nanoelectronics, molecular medicine, energy conversion, and storage [2]. These R&D have developed new opportunity areas, to create intelligent systems to solve problems based on human intelligence. This can be possible with *artificial neural network* (ANN) which is inspired by the human brain-learning activity, with the objective of solving classification problems. The application areas of ANN in nanotechnology involve classification, diagnosis, monitoring, process control, design, scheduling, and planning, and so on [3]. According to [4], ANN is the most powerful solution in sensors pre-processing information; these types of network can adapt their behavior without previous knowledge of a particular sensor response. For these cases, training algorithms are used to achieve the expected input–output relationship, by adjusting their weights in an optimal solution [4], where the input training/testing data comes from sensors that can be nano-sensors.

With the advance of nanotechnology, it is possible to manufacture nano-sensors in the scale of a few hundred nanometers. These nano-sensors have the same capability of sensing as conventional macro-sensors [5]. This progress increases the information handled by the nano-sensors, causing the need for communication between them, expanding capacity and applications in terms of complexity and range of operation. However, it requires high computational effort and therefore it is necessary to implement efficient signal processing and classification algorithms [6].

On the other hand, since the introduction in 1997 of smart mobile phones with cameras, touchscreen displays, internet connectivity, and powerful *central processing units*, (CPUs) have gained market acceptance. Presently, it is estimated that ∼7.5 billion mobile phones are in use, with 51% of them classified as smartphones, and there will be an expected increase of ∼76% by 2022 [7]. Smartphones have many types of sensors such as *global positioning system* (*GPS*), temperature, microphone, camera, humidity, compass, gyroscope, barometer, and accelerometer; those sensors are actually constructed by nanotechnology, so classification algorithms can get more information to improve their performance; however the main classification algorithm remains the same.

The smartphone sensors allow the sampling of raw data to obtain information about human mobility or activities of human behavior [8]. One of the activities that can be studied by the collected data is the behavior of a driver who is currently using his smartphone while driving, which can lead to a car accident. The average time eyes are taken off the road when texting while driving is 5 s; if this period of time is taken at 55 miles per hour, then a distance equal to a football field length will be driven without looking at the road. Because of that, the chances of suffering a car accident increases up to 23 times when texting and driving [9].

In the United States, it is forbidden to use hand-held cell phones while driving in 12 states; 37 states ban cell phone use for novice drivers; 20 states ban the use of cell phones for school bus drivers; 41 states, D.C., Puerto Rico, Guam, and the US Virgin Islands ban texting for all drivers, ref. [10]. To avoid being caught holding a cell phone and avoid a fine, drivers use the phone between their legs, causing a major distraction when using it and taking the eyes off the road for a longer period of time.

Therefore, a classification system to detect whether a driver is using his phone or not while driving is proposed in this paper as an entire application. The development of this application was done in Mexico City, where it was found that 20% of traffic accidents are caused by using smartphones while driving according to the *Mexican Association of Insurance Institutions* (AMIS, its acronym in Spanish). The metropolitan transit regulation in Mexico City sets out in Article 6, Section XI that it is prohibited to hold telecommunications equipment or other objects that may constitute a distractor for safe driving [11].

Despite all the information provided by the Mexican government on the risks of using cell phones while driving, the transit regulations applied to this topic and the increase of accidents due to this activity, the use of cell phones while driving is still a growing trend. To detect or avoid this practice, several solutions are available in the market, but either they require user interaction to start functioning or require the installation of specific hardware inside the car.

The main objective of this paper is to present a novel training metaheuristic algorithm for ANN, offering the feasibility to implement ANN for mobile applications in an optimized way. In addition, experimental engineering applications are implemented to evaluate and explain the proposed algorithm.

This work shows the relevance of optimized ANN in classification applications. First, an application where the ANN are implemented for logic gates classification is presented, where the main contribution lies in the proposed training method, which is achieved through an *earthquake algorithm* (EA). This classification study is a critical issue in nanotechnology applications where synthesis of logic circuits its needed, such as the example shown in [12], where a reversible binary-coded decimal adder/subtractor was designed and later optimized using a *genetic algorithm* (GA). Also, in [12], the relevance of synthesizing logic circuits in nanotechnology, quantum computing, and low power *complementary metal-oxide semiconductor* (*CMOS*) designs is highlighted.

**Figure 1:**
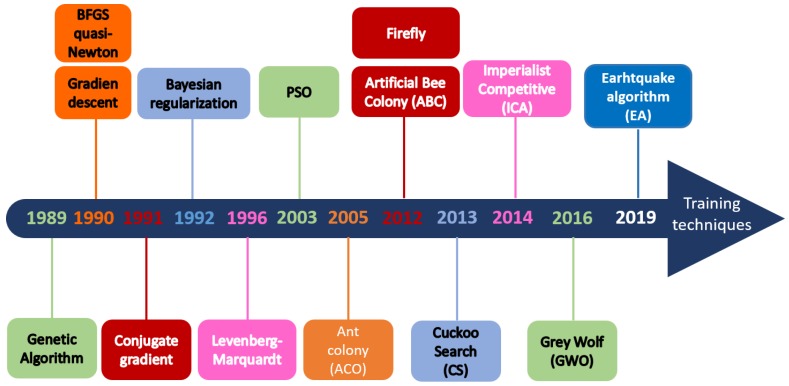
Back-propagation techniques.

Since back-propagation was developed by Werbos in 1974 and rediscovered by Parker in 1985, many optimization methods were implemented for the training process, [13]. Figure 1 shows a timeline in which it can be seen that in recent years, metaheuristic methods are used due to reduced computational effort and enhanced methods to find optimal solutions.

After validating the proposed training algorithm for ANN, a more complex classification problem is presented. The detection of users that are distracted while driving and the detection of the action of texting while driving, are real concerns in preventing automotive accidents. The application shown in [14], where effect is shown of drivers texting and eating on vehicle dynamics, is a clear example of the relevance of this issue. Nevertheless, the method showed an 86% maximum achievable precision for detecting users texting while driving.

In [15], a mobile application that uses *automatic speech recognition* (ASR) is suggested to answer to incoming text messages. The application identifies certain phrases spoken by the user and sends predefined answers according to the phrase registered. The phone reads the incoming messages to the user.

The use of the three-axis accelerometer integrated on the phone is proposed in works such as [16] and [17], where the first one has as main purpose of identifying sudden lane changes, or sudden change in the car’s acceleration or irregularities on the road, but does not take into consideration if the driver is distracted or not by the phone.

On the other hand, ref. [17] seeks to acquire acceleration data from the phone inside the car, to compare it to values parametrized to normal driving, and also to values characterizing drunk-driving users. The proposed application is shown to be able to determine if the actual driving corresponds to drunk driving, regardless of the phone position inside the car. Nevertheless, this application needs to be started by the user, which can be an issue for actual drunk users.

To address the texting-while-driving issue, ref. [18] shows the autonomous detection of distracted driving by measuring the typing speed and accuracy on a phone with Symbian Operating System. Then, Shannon Entropy is used to measure the performance of texting while driving for both tasks competing for some of the same cognitive resources. This entropy is high when texting and driving, and low when texting or driving is the only action. Ref. [19], a patent registered in the United States, shows the registration of a master phone inside the vehicle proposition, where any other phone inside the car will be registered as a slave phone of the master phone, which establishes that when the speed measured by the master phone exceeds a predefined threshold, the master phone cannot be used but slaves can, allowing passengers in a particular vehicle or in a school bus to use their phone while the vehicle is in motion but preventing the driver from doing it. However, the proposed technique can be manipulated, as the driver could have two phones and register one as the master phone and the other one as a slave.

Also another patent, shown in [20], and registered in the US on 23 April 2013, proposes another mobile application capable of detecting when the vehicle speed has exceeded a speed threshold, blocking the mobile applications capable of sending or receiving data or phone calls.

In [21], a patent registered in the US on 19 March 2013, a system is proposed to disable texting functions when the cell phone enters the vehicle by the driver’s door. Once the cell phone leaves the vehicle by the driver’s door, the functions of text messages are restored. This system requires the installation of specific hardware on the driver’s door to detect if the phone enters by that door or not.

Meanwhile, the proposed solution shows how an ANN trained by the EA can be used for the task, validating with a real case study the capabilities of ANN when the correct training is made. This method is based on detecting whether the user interaction with the phone has changed instead of detecting if the user’s driving has been affected. This is achieved by measuring the acceleration experienced by the phone when certain applications are being used, where the data collected are processed by an ANN that will determine if this acceleration values correspond to values generated when texting while driving or not. To double-check the output of the ANN, the phone’s GPS is activated to determine the speed at which the phone is moving.

This paper is organized as follows: Section 2 explains the ANN training basics, then Section 3 presents the *earthquake algorithm* (EA) that adapts the principle of propagation of geology waves P and S through the earth material composed by random density to ensure the dynamic balance between exploration and exploitation in order to reach the best solution to optimize complex problems. Then, the use of EA to validate the algorithm for the training process is presented, where the AND and OR logic gates were used as a comparative for validating results. Section 4 describes the development of mobile phone usage detection while driving, showing the viability of ANN for sensor characterization and implementation. Finally, Section 5 discusses the results and Section 6 concludes the paper.

## 2. Artificial Neural Network

ANN seek to emulate the way in which the human brain processes information. ANN are biologically inspired computational models formed by artificial neurons interconnected with coefficients, which constitute artificial neural structures [22].

It is also known that the ANN are non-linear mapping systems that use processing units called neurons, where each neuron receives inputs from other nodes and generates a scalar output. The output directly depends on the weighted information taken from the inputs [23].

The general components of an ANN can be summarized as follows:

- Set of neurons.
- Connections between neurons.
- Activation functions for each neuron.

**Figure 2:**
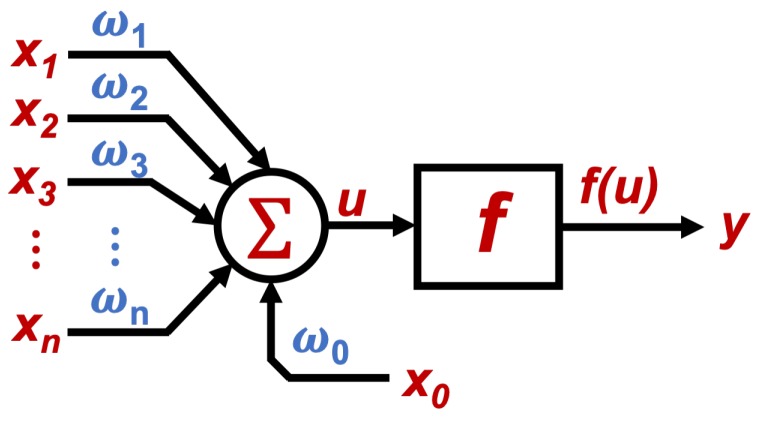
Single Neuron with multiple weighted inputs.

The connection between neurons need to be regulated with weight values to be processed, where after a weighted sum, an activation function is required to obtain the output value from the neuron input set. The structure of single neuron with *N* number of inputs *x* and weights (ω) is shown in Figure 2, where according to [24] the neuron output is given by the weighted sum described in (1).

(1) $$u=\sum_{i=1}^{n}x_{i}\omega_{i}+\theta =x_{1}\omega_{1}+x_{2}\omega_{2}+x_{3}\omega_{3}+\ldots +x_{n}\omega_{n}+x_{0}\omega_{0}$$

where *u* is the output of the weighted sum, *x* the system inputs, ω their weights and θ an adjustment parameter for the network; where $\theta =x_{0}\omega_{0}$. Then, as explained in [23], a non-linear activation function *f* is needed, in order to obtain a representative output of the system, that can be represented as shown by (2).

(2) y=f(u)

where *y* is the output of the system, and f(u) the evaluated value of *u* through an activation function. According to [23], some of the most used activation functions are:

- **Step function:** usually used when a binary output is needed.
- **Lineal and mixed function:** often chosen when the output can be taken as a linear relation from the weighted sum, in a defined range.
- **Hyperbolic tangent function:** used when smooth positive or negative variations are expected from the output.
- **Gauss function:** mainly used to reduce (when possible) hidden mapping, to one layer of neurons.

The two main ANN topologies for neuron interconnection are the feed-forward and the recurrent networks, where the recurrent networks use feed-back configuration. For the proposed ANN case study in this paper, a single layer with a single neuron using feed-forward configuration is studied.

It is important to highlight the relevance of the weights for the input processing, since they are responsible for characterizing the extent to which each input affects the system, to regulate the response in case there are two or more simultaneous input activations. Then, training the network is a process in which the weights of every input are calibrated, so that the system can match the expected behavior regarding the inputs and outputs.

The training can be done knowing weight values or feedback methods and learning patterns can be used to modify the weights between the ANN layers, until the right weights are obtained. The learning process can be supervised or not. In the supervised one, input values are introduced, and the corresponding output values are known. In the non-supervised learning, similar characteristics among the input data are tried to be found.

One of the most used methods for ANN training is the back-propagation method, which is a supervised learning method based on the relationship between input training patterns and generated outputs, which, taking the mean error minimization using the mean square error method, adjusts the network weights to emulate the expected behavior.

Learning can be defined as an iteration process, where in each iteration the weight values between layers are modified until reaching the desired output. The difference between the desired output value and the one obtained by the ANN is called error [23]. That error is propagated to the previous neurons for adjusting their weights.

## 3. Earthquake Algorithm

Earthquake is one of the most destructive phenomena, occasioning human and economic loss. When an earthquake occurs, elastic energy is accumulated in seismic regions; if the friction is overpassed then shear movements are generated originating an earthquake [25]. Over years, there have been discussions about information that can carry distinct types of waves (P and S) that compound an earthquake. The first seconds of the P-wave transports information about the final magnitude of the seismic [26]. This principle is used to make the velocity of P-wave as an explorer agent providing information from search space to acquire the optimum solution.

In [27], earth material is described by Hooke’s Law in elastic behavior [28]. Passing this elastic limit, earth material may have performances with both brittle fracturing and ductility. According of the deformation type, elastic and earth material could be quantified by elastic moduli mentioned in [29].

**Figure 3:**
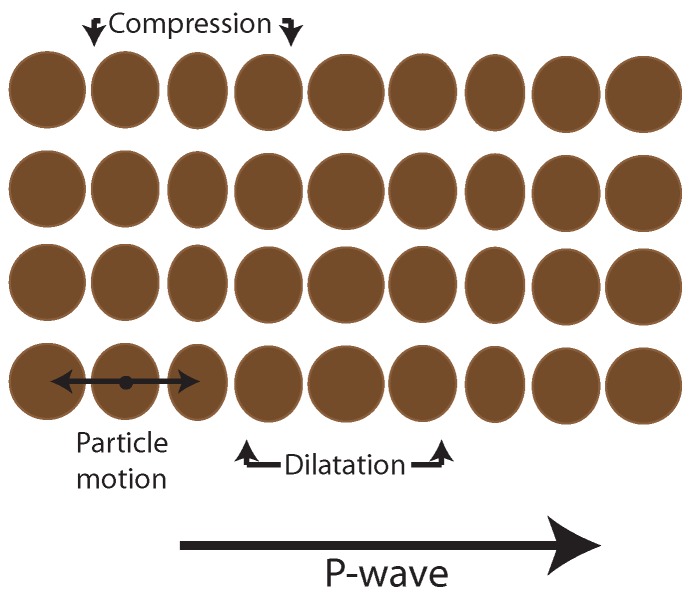
P-wave movements (compression and dilation).

**Figure 4:**
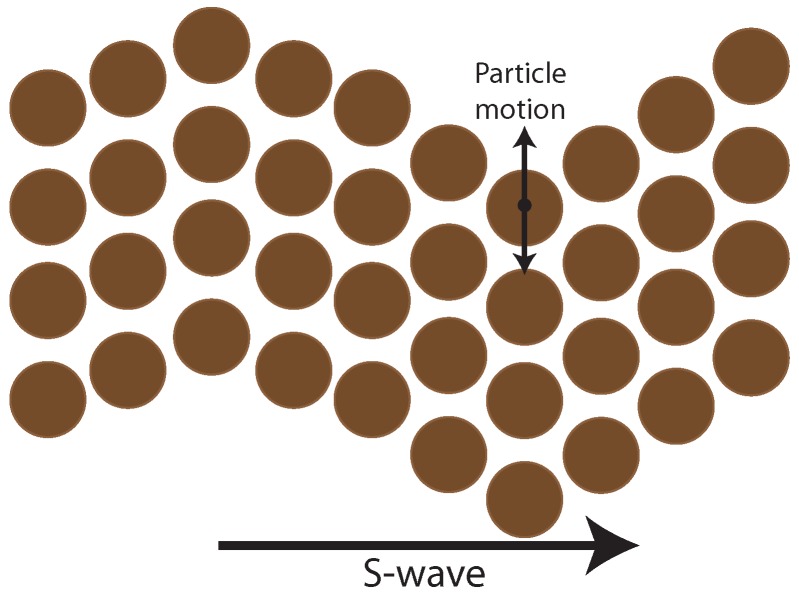
S-wave movements by shearing deformation.

Two types of waves, P and S, are generated. The P-wave is the fastest and depends on the earth material compressibility (Figure 3). S-wave is slower than P-wave and depends on rock elasticity causing epicenters to move up and down, perpendicular to the wave direction (Figure 4).

The *Earthquake Algorithm* (EA) is a geo-inspired algorithm, based on the P and S waves existing in earthquakes, similar to as proposed in the first version developed in [30]. Then, as explained in [31] Equations (3) and (4), show the mathematical expressions used for the P and S waves velocities.

(3) $$v_{p}=\sqrt{\frac{\lambda +2\mu }{\rho }}$$

(4) $$v_{s}=\sqrt{\frac{\mu }{\rho }}$$

where $v_{p}$ and $v_{s}$ are the speed of waves, λ and μ the Lamé parameters, and ρ the density of material.

According to [29], the Lamé parameters can be the same under some circumstances, so for the current algorithm it is taken that λ = μ. In that case, to find the optimal Lamé parameters to be used, several tests were performed with different Lamé values, finding that the only real constant that worked was 1.5, therefore:

(5) λ=μ=1.5GPa

On the other hand, for the implementation of the algorithm, the density of the solids (ρ) is used as a random value, selected from a range between 2200 and 3300 Kg/m${}^{3}$, also according to [29].

**Figure 5:**
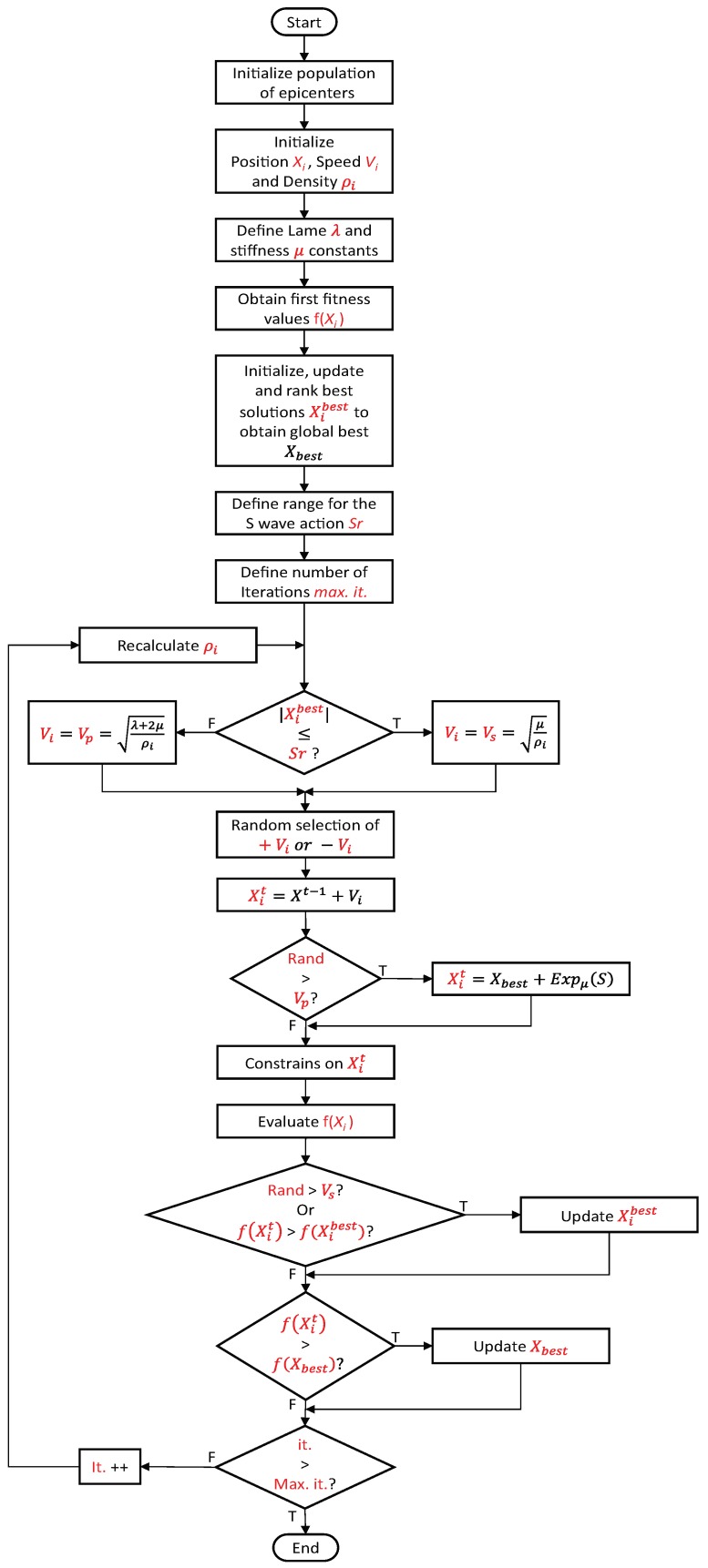
General architecture for *EA*.

Figure 5 visualizes the general diagram that describes the earthquake algorithm flow, where, in order to determine when to use $v_{p}$ or $v_{s}$, it is essential for the algorithm to define an operation range for the S wave, which will be referred to in this document as the S range or Sr.

The Sr should be assigned with previous knowledge of the problem needs; nevertheless it is recommended to implement the range in the function of the percentage of error, between the obtained solutions and the expected ones.

Therefore, the Sr is recommended to be 2% from the best solution, but after selecting if the current epicenter is going to use the $v_{s}$ or $v_{p}$ as $v_{i}$, its results are very important for the algorithms performance to understand, since both speeds are calculated by a square root. The final result is a positive number, but it is also known that the result contemplates a $v_{i}$.

The above is the reason that the flowchart that describes the proposed architecture of the algorithm contemplates the use of a random selection of a positive or a negative $v_{i}$, just to give to the EA another degree of freedom. As another option to visualize the operating principle of the algorithm, Algorithm 1 shows the pseudocode of the EA.

**Algorithm 1** Pseudocode of the EA. 1: **Define**
*objective function*. 2: **Initialize** population of *epicenters*. 3: **Randomly initialize**
*x${}_{i}$*. 4: **Initialize**
*v${}_{i}$* and $\rho_{i}$. 5: **Define**
μ and λ. 6: **Obtain and rank** the first *fitness values*. 7: **Initialize**
*x${}_{i}^{best}$* and *x${}_{best}$* with the *fitness values*. 8: **Define**
*Sr*, and the *Max. it.* 9: **while** (*it.* < *Max. it.*) **do** 10: **Randomly calculate**
$\rho_{i}$. 11: *v${}_{p}$*←$\sqrt[{}]{\frac{\mu }{\rho_{i}}}$ 12: *v${}_{s}$*←$\sqrt[{}]{\frac{\lambda +2\mu }{\rho_{i}}}$ 13: **if** (*∣x${}_{i}^{best}|$*≤ *Sr*) **then** 14:  vi←vs 15: **else** 16:  vi←vp 17: **end if** 18: **Randomly select** positive or negative *v${}_{i}$*. 19: $x_{i}^{t}\leftarrow x_{i}^{t-1}+v_{i}$ 20: **if** (*rand* >*v${}_{p}$*) **then** 21:  $x_{i}^{t}\leftarrow x_{best}+Exp_{\mu }\left(s\right)$ 22: **end if** 23: **Constrain**
*x${}_{i}$* if needed. 24: **Obtain** the new *fitness values*. 25: **Actualize** x${}_{best}$ and x${}_{i}^{best}$ 26: *it.← it.++* 27: **end while** 28: **Postprocessing and visualization** of results.

**Figure 6:**
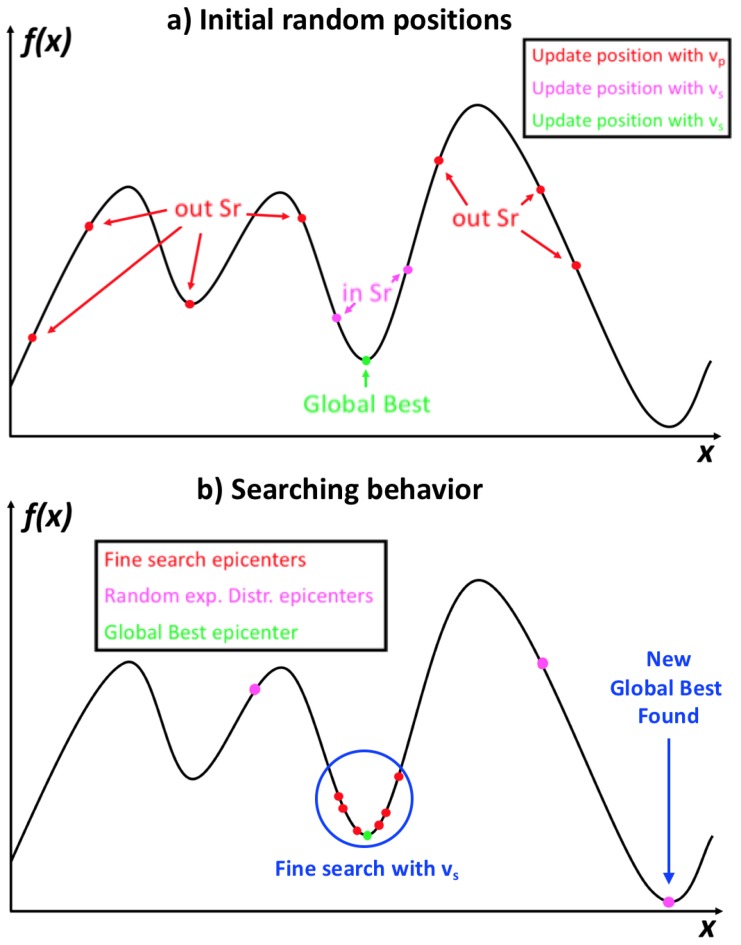
Behavior of the earthquake algorithm using 10 epicenters.

To clarify how the proposed algorithm works, Figure 6 shows the generalities of the earthquake algorithm behavior, where Figure 6a shows an example of how the epicenters could be randomly placed on a function, as when evaluated using the first fitness values.

After the first rank of the best solutions is obtained, the epicenters that are in or out of the *Sr* can be known, and hence, those that are going to use the $v_{s}$ or $v_{p}$ speed to update their positions will also be known. Also, the *Sr* is pointed out in Figure 6b, highlighting the concept of the epicenters near to the current best solution take $v_{s}$, as searching speed.

Also, in Figure 6b, it can be seen that after some iterations, the epicenters start to converge with a fine search around the current global best. Despite this, it can be observed that three epicenters took distant routes from the epicenters set because of the random generation of positions using the exponential distribution (previously explained).

The fact that the final generation of some epicenters allowed the algorithm to escape the local minimum resulted in another epicenter finding a better solution. Replacing the previous global best solution, the epicenters would converge again to begin another fine search, retaining the possibility that another randomly generated epicenter finds another “best solution”.

### ANN Training Using EA

By the fact that a training technique that works for single-layer ANN can be extrapolated for most of the existing topologies, two cases were selected to validate the viability of the EA for the training process; the first one is an AND logic gate, and the second an OR logic gate.

As is already known, both logic gates expect a logic value (0 or 1) as output, because a step activation function will be used for the neural networks in this paper.

Logic gates are widely used in an infinite variety of areas, but it is known that there are only three main logic gates, and the others can be obtained by combinations between them:

- **AND:** Logic gate that is activated when all its inputs are in true state.
- **OR:** Logic gate that is ON when any of its inputs are in true state.
- **NOT:** Logic gate whose output is always inverted from the input.

Then, in order to prove the capability of the EA for ANN training, two basic cases are proposed: a single perceptron that works as an AND, and a single-layer perceptron working as an OR gate. Table 1 shows a summary of the expected outputs regarding to the inputs of both logic gates.

**Figure 7:**
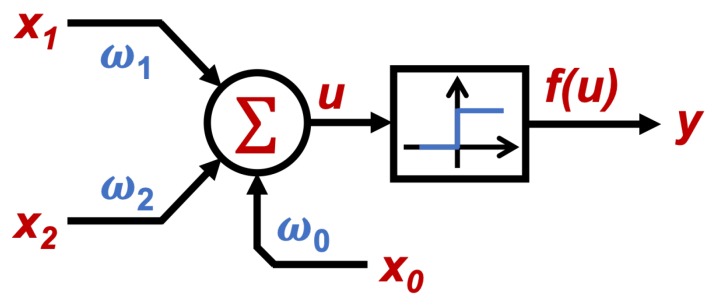
ANN topology selected for both cases.

**Figure 8:**
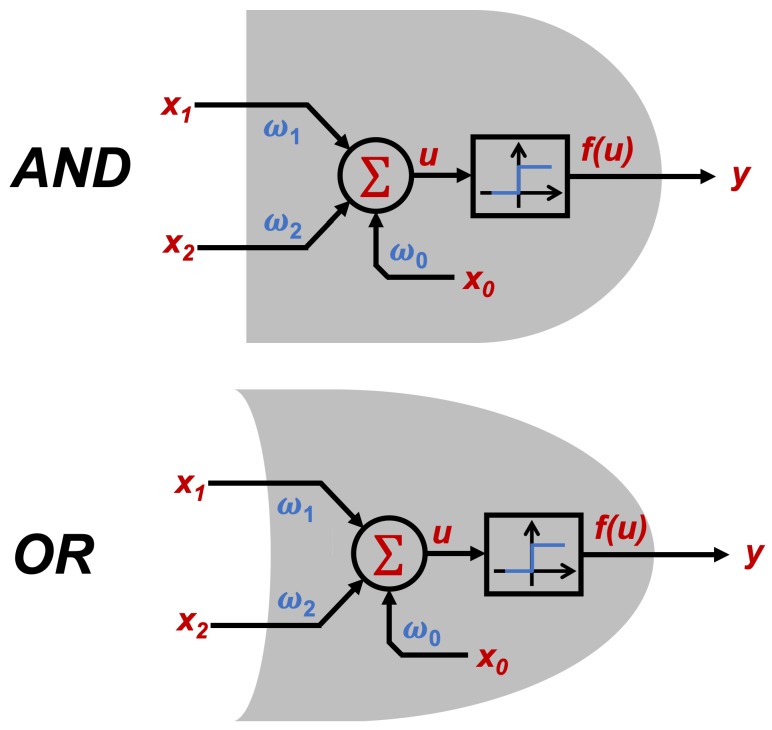
Logic gates AND and OR using ANN.

Regarding the ANN topology to be used, a single-layer perceptron network will be evaluated, since as shown in the truth tables shown in Table 1, both cases are linearly separable. Figure 7 shows the diagram of the topology, and Figure 8 illustrates how, implicitly, the results of the networks implemented is the emulation of the behavior of the logic gates.

Then, to understand what to expect as the training result from the EA, Section 5.1 shows the linear separation of the inputs that may activate or remain inactivated, and the gate outputs.

A back-propagation technique is taken for error evaluation, to evaluate how the error acts after the non-linear activation function. Where the EA was implemented using 10 epicenters, for 10 iterations as a maximum number of iterations and using normalized initial weights set for the epicenters.

**Figure 9:**
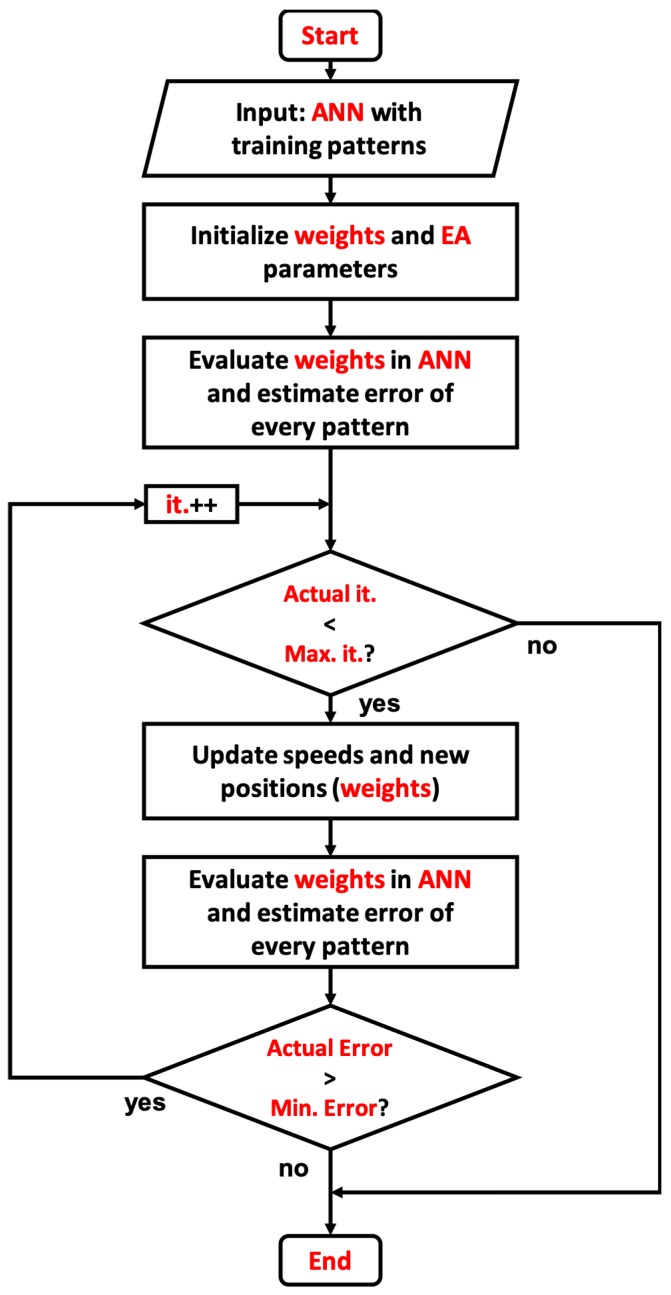
General architecture for ANN training through *EA*.

Figure 9 shows the general structure of the ANN training technique based on propagation of the error, but this time using the EA optimization technique. The input of the system should be the training patterns that the network should emulate or learn, the weights are the optimization variables that are going to use as positions for the algorithm, while the evaluation process is made by the response of the ANN with the selected weights with every input learning pattern. The cost function is taken as the error between the expected output against the obtained value.

Meanwhile, the main loop has two conditions—exit by exceeded iterations or by the minimum error achieved. The update velocities and positions are made by the EA $V_{P}$ and $V_{s}$ equations, therefore the optimization algorithm works with the back propagation of the error in order to achieve the pattern emulation.

Finally, the flowchart shown by Figure 9 is summarized by the pseudocode shown in Algorithm 2, where it can be clearly seen how the weight optimization can be applied using the EA. Nevertheless, it is important to highlight that the actual ranking of the solution is made by selecting the response with less error after the selected ANN topology is evaluated with the weights. The learning process is achieved after the maximum iterations are reached, or when the error is less or equal to the required error; for the logic gates tests, the selected error was equal to 0 in order to achieve a solution that completely achieves the task.

**Algorithm 2** Pseudocode of the EA for ANN training process. 1: **Define**
*Training patterns for the ANN*. 2: **Initialize** population of *epicenters (weights for the ANN)*. 3: **Randomly initialize**
*x${}_{i}$*. 4: **Initialize**
*v${}_{i}$* and $\rho_{i}$. 5: **Define**
μ and λ. 6: **Obtain and rank** the first *fitness values through the ANN results*. 7: **Initialize**
*x${}_{i}^{best}$* and *x${}_{best}$* with the *fitness values*. 8: **Define**
*Sr*, and the *Max. it.* 9: **while** (*it.* < *Max. it.*) **do** 10: **Randomly calculate**
$\rho_{i}$. 11: *v${}_{p}$*←$\sqrt[{}]{\frac{\mu }{\rho_{i}}}$ 12: *v${}_{s}$*←$\sqrt[{}]{\frac{\lambda +2\mu }{\rho_{i}}}$ 13: **if** (*∣x${}_{i}^{best}|$*≤ *Sr*) **then** 14:  vi←vs 15: **else** 16:  vi←vp 17: **end if** 18: **Randomly select** positive or negative *v${}_{i}$*. 19: $x_{i}^{t}\leftarrow x_{i}^{t-1}+v_{i}$ 20: **if** (*rand* >*v${}_{p}$*) **then** 21:  $x_{i}^{t}\leftarrow x_{best}+Exp_{\mu }\left(s\right)$ 22: **end if** 23: **Constrain**
*x${}_{i}$* if needed. 24: **Obtain** the new *fitness values*. 25: **Actualize** x${}_{best}$ and x${}_{i}^{best}$ 26: *it.← it.++* 27: **end while** 28: **Postprocessing and visualization** of results.

## 4. Mobile Phone Usage Detection while Driving

This section seeks to present an experimental implementation for ANN, where a novel concept that enables Android-based cell phones to autonomously detect the usage of a cell phone while driving is detailed. The case study is achieved by measuring the acceleration experienced by the phone when certain applications are being used, through the accelerometer sensor of an Android cellphone. The collected acceleration data are later processed by an ANN that will determine if the current acceleration corresponds or not to data generated when the user was texting while driving.

To validate the results, ANN processed data from the accelerometer, and the GPS sensor is used to determine the speed at which the phone is moving in all the tests, demonstrating the reliability of the data obtained.

### 4.1. Theoretical Background

#### 4.1.1. Accelerometer on Android©

**Figure 10:**
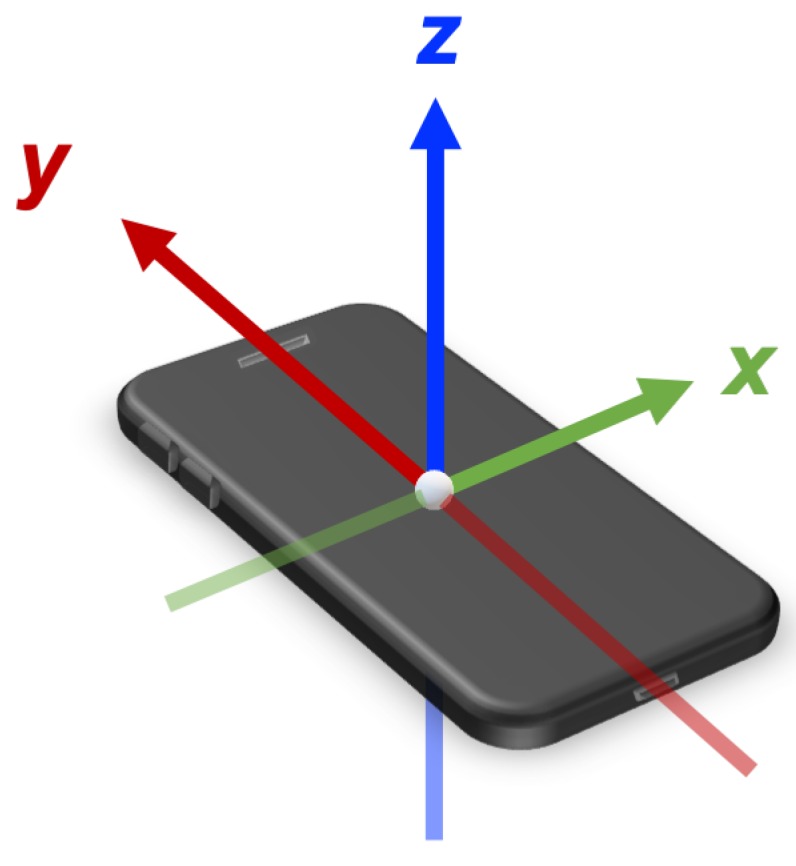
Coordinate system of the phone in default position.

As already detailed in [32], the accelerometer in Android phones works through events or interruptions, imposing in some cases heavy limitations regarding sampling rates, where the measured value is the acceleration experienced by the phone in m/s${}^{2}$ plus the acceleration because of gravity. The coordinate system is defined for this work relative to the screen of the phone when it is in the default orientation, where the x-axis is horizontal and has positive values to the right, the y-axis is vertical and has positive values upwards, and the z-axis is positive when pointing away from the phone display [33]. The described coordinate system is shown in Figure 10.

To be able to read the information collected by the accelerometer, the minimum Android level must be 1.5, which is *application program interface* (API 3). Nevertheless, as explained in [34] the noise from the sensor measure must be taken into consideration for the final processing of the collected data from the accelerometer. For this work, a simple programmed digital filter is applied, seeking to clean the objective features from the measured noise.

After the data acquisition from the accelerometer, the speed from the vehicle can be estimated by integrating the acceleration resultant curve, where, as shown in [35], the *x* and *y* axis components are the critical data to correctly estimate the speed of the vehicle. The data from the *z*-axis did not have a relevant or useful impact in the measurements, just as expected, since the displacement of the driver is only significative in the horizontal plane created by the *x* and *y* positive and negative axis. Also, as explained in [35]’s method, the data from the accelerometer can be used to understand and analyze the driver’s behavior in relation to the speed data, a fact that would later lead to find if the driver is texting while driving or not.

#### 4.1.2. GPS on Android©

According to [36], the GPS has critical importance for enabling and improving positioning performance in smartphones. Particularly for Android mobile phones, the data acquisition can be made from the on-board chipset and through Android’s *Application Programming Interface* (API), also as described in [36].

Android provides the option of using the GPS in two ways. The first is through the use of satellites and allows a fine location. The disadvantage of this is that to operate the phone must have a direct visibility of the satellites. The second option for the location is based on information received from cell phone towers or Wi-Fi access points; its access is based on the network. This option works even inside buildings if the phone has network service [37].

For security reasons, access to the location of the phone is restricted to applications. If an application needs to know the location it must request this permission to the user. To do this this, permission must be specified in the *AndroidManifest.xml* file. It is recommended to seek permission for both methods of localization for the system to operate even when one is not available. The permissions required are:

- android.permission.ACCESS_COARSE_LOCATION
- android.permission.ACCESS_FINE_LOCATION

The accuracy of this type of speed estimation method using GPS was demonstrated in [38], where it is also shown that it can be used as a reference speed for other sensor calibration, and later can be used for a speed model taken by the average distance traveled during a certain amount of time. These types of method using the correlation between the acquired data of two different sensors improve the accuracy of the results, as described in [39] and [35] applications.

### 4.2. System Design and Implementation

**Figure 11:**
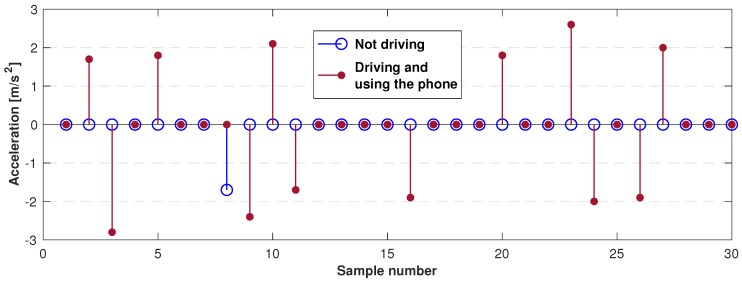
Comparison between the acceleration of the two groups.

**Figure 12:**
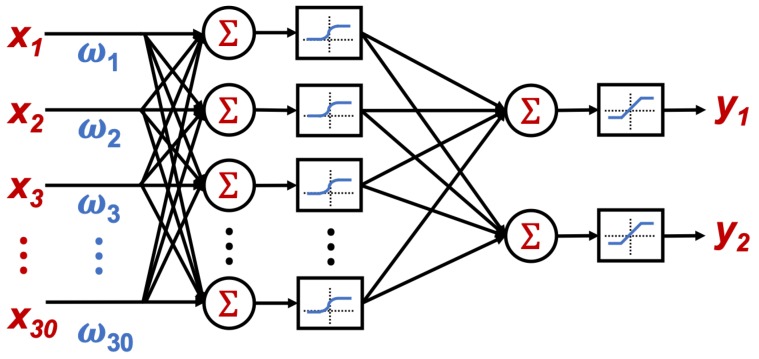
Proposed artificial neural network architecture.

**Figure 13:**
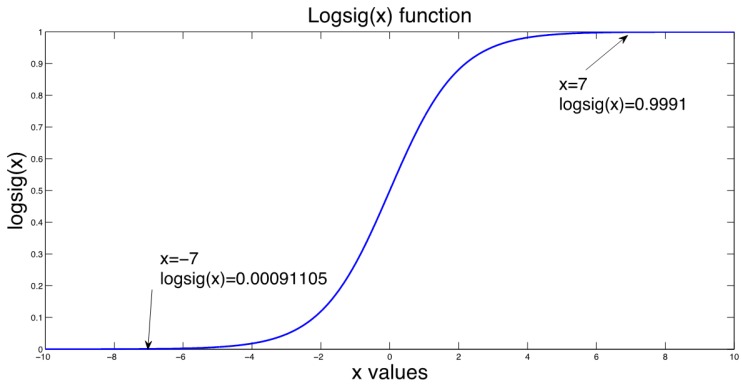
Sigmoid activation function.

The system was implemented on an Android phone with the following characteristics. The system design relies on four basic stages, which are:

1. Detect unusual behavior when using the phone: The proposed ANN classification for the task in the app must be able to run in the background; therefore, it must be implemented as a service. In this service, the application reads the acceleration values registered by the phone, the acquired values are not continuous data to reduce battery consumption. The data from the acceleration is only acquired when several applications are being used, where the applications that will be monitored to detect their use are summarized in Table 2.
  If any of the applications in Table 2 is in use, the phone accelerometer is turned on to register acceleration values and determine if the driver is using the phone. This classification is made using an artificial neural network which is trained by the EA described above.
  To train the artificial neural network, vectors containing 30 acceleration samples each were used. Two groups of data with similar characteristics on each group were defined. One group corresponds to the acceleration values when using the phone while driving, and the second group corresponds to the acceleration values when doing anything else. To obtain these samples as close as possible to reality, they were collected on a real texting-and-driving scenario. The acceleration values experienced by the phone were recorded while the car traveled between 10 and 100 km/h and the driver was using the phone. In total, more than 780 acceleration vectors were collected.
  The collected acceleration values are recorded in their positive and negative values. The difference between the two groups can be seen in Figure 11.
  Once the ANN was trained and the weights and bias values obtained, the obtained network is a feed-forward ANN with back-propagation with 30 neurons in the input layer, one hidden layer with 30 neurons and a sigmoid activation function and finally, the output layer with two neurons and linear activation function as shown in Figure 12.
  Once the artificial neural network has been trained, it was implemented on the mobile phone. To perform the portability of the neural network to the cellphone, the resultant weight and bias values were stored in arrays, where the activation function sigmoid was also stored in an array by taking its resultant values in the interval from −7 to 7. Therefore, to evaluate the function for a given value, the index from the array of the value must be looked-up to get the result. The selected range for the look-up table (−7 to 7), was taken by the fact that the proposed sigmoid function values correspond almost to saturation as seen in Figure 13.
2. Determine if the phone is experiencing speed: After the unusual behavior when using the phone detection, if the artificial neural network determines that the input data is classified into the group of driving and using the phone, the GPS is turned on to verify the speed of the phone. The GPS was set to report location updates using a 500 millisecond sampling time, and updating the distance every single traveled meter.
3. Notify the user: The next step to be followed is if the GPS determines that the phone has speed and it is greater than a threshold speed (15 km/h), the phone screen must be locked and the event must be reported to a database in order to quantify them.
  On the other hand, if the phone does not have speed or the speed is less than the threshold speed, the application must turn off the GPS and do nothing else.
  For this task, the application must have administrator permission to lock the screen whenever it detects the use of the phone while driving. This permission must be requested by the application on start, and later granted by the user.
4. Notify the event to a higher entity: Finally, the information is sent to the database by a request of type Post, where the sent data corresponds to the date and hour when the event took place, the speed registered, and also the location with latitude and longitude where it happened.
  This information is stored in a table inside the database, where the data modification inside the table is done via a *.php* file stored in the domain.

**Figure 14:**
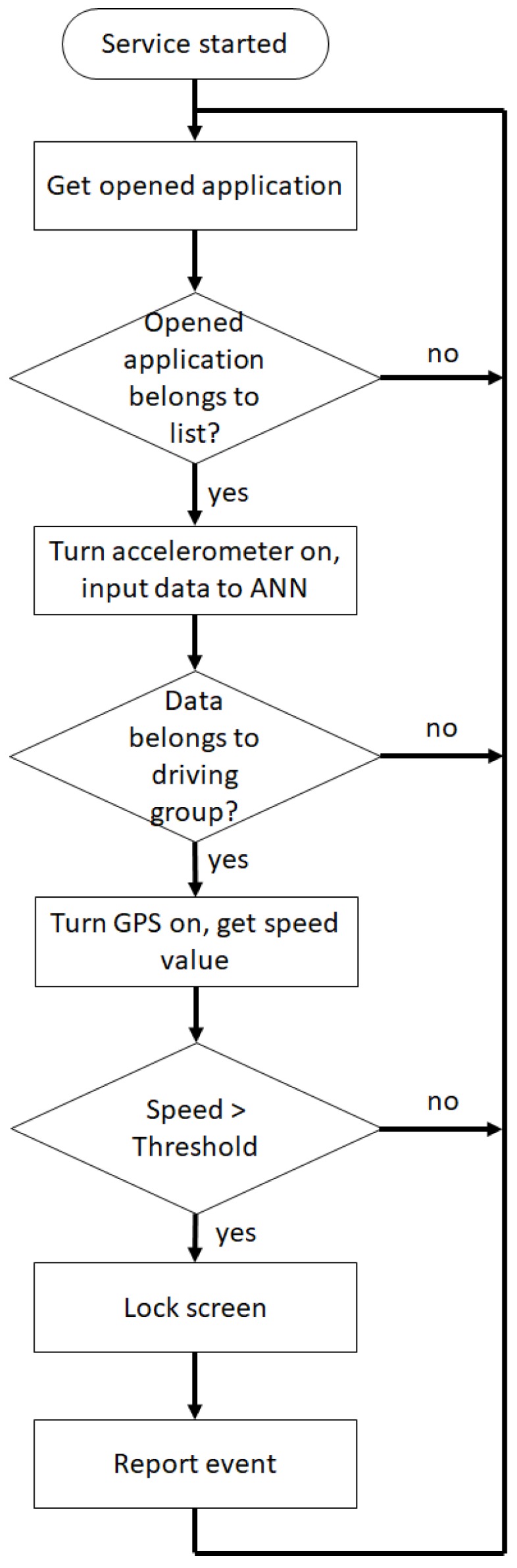
Architecture of the system detection algorithm.

Those described stages completely describe how the detection of a used phone is made, and the actions taken after the events; nevertheless, the algorithm that summarizes the proposed method is highlighted by Figure 14.

## 5. Results

The following results present the capability of the EA to adapt for different optimization study cases, where first the ANN training results are presented, and later the mobile phone usage detection while driving results are analyzed.

### 5.1. ANN Logic Gates

As previously explained in Section 2, the AND and OR logic gates are linearly separable sets, because the single-layer perceptron topology was selected for the classification, where the Earthquake Algorithm performed the weights adjustment, to emulate the selected behavior.

The EA was run 15 times in order to achieve an average final error evaluation, and also to obtain a mean value for the iterations it took to solve the ANN weight training. Nevertheless, the results showed that the final error for the 100 times was 0, and that on average it takes 1 iteration to achieve the task for both gates emulation.

**Figure 15:**
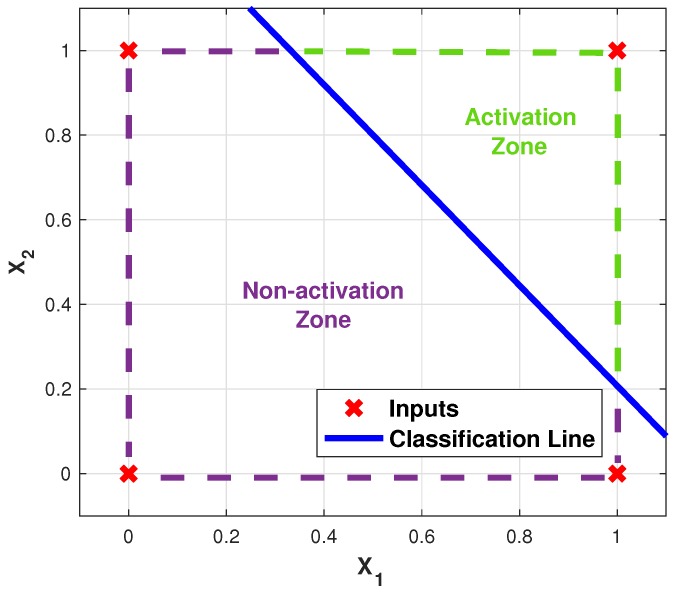
ANN emulating the AND logic gate response.

Table 3 shows the mean values of the weights obtained by the earthquake algorithm, for which final behavior can be seen in Figure 15, where it is graphically shown that in the activation zone it is only the case where both inputs are activated, just as happens in a typical AND logic gate.

**Figure 16:**
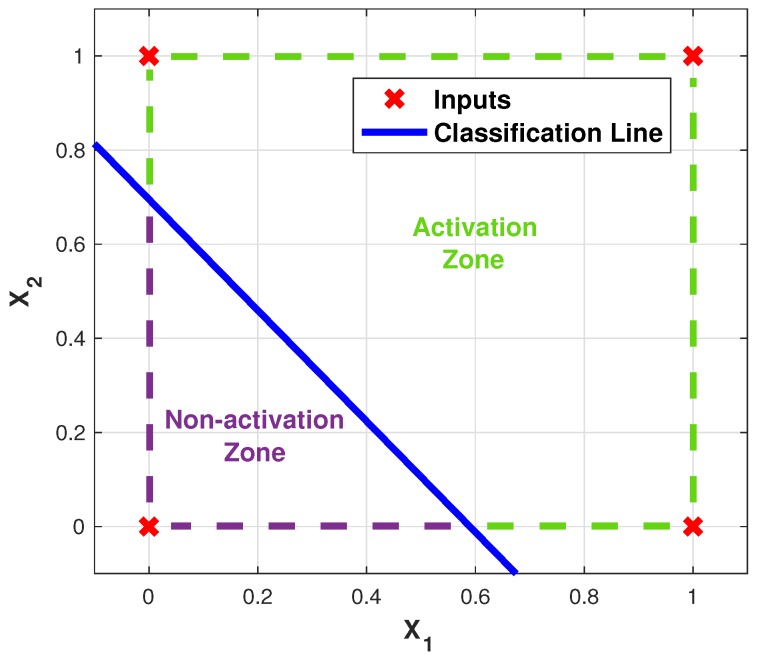
ANN emulating the OR logic gate response.

Finally, Table 4 shows the mean values of the weights after the training process, whose final behavior is shown in Figure 16, where it can be seen that in the non-activation zone is only given by the case where both inputs are not activated, just as happens in a typical OR logic gate.

Then, as already explained, the ANN AND and OR gates can be used to extrapolate the results, to obtain other logic gate topologies and even to train other ANN for different applications, where most of the time, the main objective function is going to be related to the output expression that relates the inputs with the weights and the expected output values.

### 5.2. Mobile Phone Usage Detection

When validating the response of the artificial neural network, Table 5 shows the results, which were obtained according by the evaluation of different membership percentages.

As can be seen in Table 5, the percentage of success of the network increases when the membership percentage decreases. The membership percentage set on this project was >0.60 because as there are only two groups, if it does not belong to one it must belong to the other.

After setting the selected membership percentage, the network was tested on the phone, setting it to turn on GPS whenever the network result indicates the driver is using the phone, and not doing anything when the network determines the user is not driving. The success percentages of detecting if the user was driving or not are shown in Table 6.

As demonstrated by Table 6, the application can determine when the user is not driving or inside a car in every situation. During the performed tests, the application never locked the screen when the user was not in a car but doing any other activity while using the phone.

On the other hand, the driving and using phone success of the application was 96%. The failed events detection in the app are mostly related to the acquired skills of the test driver to use the phone while driving, which in some cases added some uncertainty to the data collected for the training process.

Every time the application detected, and the driver was using the phone, the screen was locked and the event was reported to the table in the database, in order to quantify the detected events at the speed they occurred. The uploaded events data is displayed in Table 7.

During the tests taken it was observed that the application detects not only when the driver uses the phone but also, not in every event, when a passenger does. This is an undesired response of the application for an objective that was to lock the screen only when the driver uses the phone and to allow the other passenger to freely use theirs.

The battery consumption of the application is low, even when it was tested for more than 120 h continuously and still did not represent more than 5% of battery consumption. Currently, the application has been registered in Mexico’s National Institute of Copyright under the following registration number: 03-2013-110709325500-01.

As a future work the authors want to make the app run only when the screen is on, to consume even less battery. Also, the app must detect when the user is dictating to the phone, and must not lock the screen considering that it does not distract the operator’s field of vision. Additionally, the user should be able to select which applications can be set as monitored applications, regarding the constant appearance of new applications and new social networks that could be a distraction.

**Figure 17:**
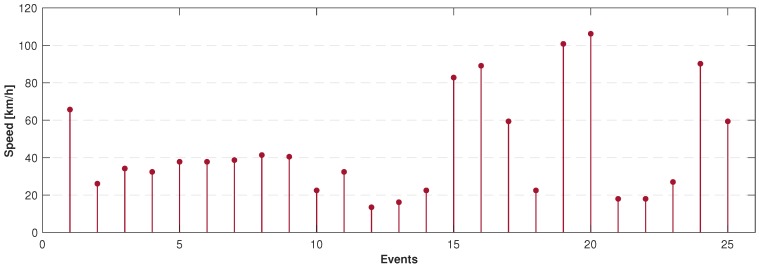
Speeds during detected events.

**Figure 18:**
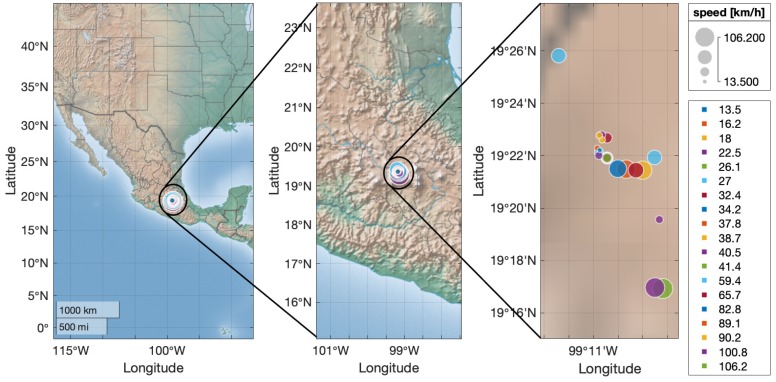
Geo-localization of the detected events.

Finally, looking again at the detected events from Table 7, Figure 17 shows the plot of the speeds from the detected events, validating that the ANN is clearly respecting the threshold limits proposed for the detection. Additionally, the geo-localization of the events is shown in Figure 18, where the place where the events happened can be tracked with the latitude and longitude, showing also on the map the presented speed in every case.

## 6. Conclusions

The Earthquake Optimization Algorithm proved to be suitable for ANN training, or even sensor information classification. Nevertheless, it showed remarkable results in terms of computational cost for the neural network training process. The convergence of the algorithm was also tested in the model optimization, where different velocity actions are shown, proving fast evolution of the error and demonstrating how the finer S-parameters allow a more accurate optimization.

Also, the case study investigating mobile phone usage detection while driving showed that the application is able to determine when the user is not driving or inside a car in every situation, but energy-harvesting techniques should be added in order to improve the performance; nevertheless, ANN capabilities for implementation techniques for sensor applications are clearly proved. The application can determine when the user is driving and using the phone 96% of the time. The application runs in the background continuously and its consumption of energy does not exceed 5% of the phone battery. The response time of the system to detect if the user is driving and using the phone is less than 3 s.

Finally, it is determined that the result of this application is successful on the phone on which it has been tested, under the driver’s driving pattern. It needs to be installed on different brands of smartphones that have accelerometers from different manufacturers. Nevertheless, the accuracy of the phone usage detection was remarkably good, demonstrating that the ANN was correctly trained by the EA, which also shows how the algorithm can be used as a training method for other ANNs.

## Figures and Tables

**Table 1:** Logic gates used for the case study.

Logic Gate	Gate Symbol	Truth Table		
AND	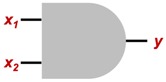	*x*_1_	*x*_2_	y
		0	0	0
		0	1	0
		1	0	0
		1	1	1
OR	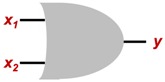	*x*_1_	*x*_2_	y
		0	0	0
		0	1	1
		1	0	1
		1	1	1

**Table 2:** Applications of interest [40].

Application	Number of Active Users
Whatsapp©	1600 million (monthly)
Facebook©	2320 million (monthly)
Facebook messenger©	1300 million (monthly)
WeChat	1098 (monthly)

**Table 3:** ANN obtained weights for the AND logic gate behavior emulation.

$\omega_{0}$	$\omega_{1}$	$\omega_{2}$
0.8296	0.7061	0.5953

**Table 4:** ANN obtained weights for the OR logic gate behavior emulation.

$\omega_{0}$	$\omega_{1}$	$\omega_{2}$
0.3386	0.5741	0.4869

**Table 5:** Validation of ANN result.

Membership Percentage	Network Response Success	
	Not Driving	Driving and Using Phone
>0.90	15.56 %	52.55 %
>0.80	27.41 %	65.69 %
>0.70	68.15 %	72.26 %
>0.60	82.96 %	78.10 %

**Table 6:** Success of entire system.

Not Driving	Driving and Using the Phone
100 %	96.18 %

**Table 7:** Detected events information.

Latitude	Longitude	Speed
19.3576	–99.1548	65.7
19.3654	–99.1741	26.1
19.3654	–99.1741	34.2
19.3654	–99.1741	32.4
19.3654	–99.1741	37.8
19.3654	–99.1741	37.8
19.3654	–99.1741	38.7
19.3654	–99.1741	41.4
19.3654	–99.1741	40.5
19.3799	–99.1777	22.5
19.378	–99.1742	32.4
19.3703	–99.179	13.5
19.3715	–99.1808	16.2
19.3667	–99.1796	22.5
19.3585	–99.1669	82.8
19.3581	–99.1613	89.1
19.3656	–99.1422	59.4
19.3259	–99.1389	22.5
19.2826	–99.1418	100.8
19.2822	–99.1365	106.2
19.3768	–99.1774	18
19.3797	–99.1794	18
19.369	–99.1796	27
19.3574	–99.1499	90.2
19.4304	–99.2069	59.4
